# Independent predictors of insulin resistance in Brazilian adolescents: Results of the study of cardiovascular risk in adolescents–Brazil

**DOI:** 10.1371/journal.pone.0246445

**Published:** 2021-02-09

**Authors:** Maria Izabel Siqueira de Andrade, Juliana Souza Oliveira, Vanessa Sá Leal, Poliana Coelho Cabral, Pedro Israel Cabral de Lira

**Affiliations:** 1 Departamento de Nutrição, Universidade Federal de Pernambuco, Recife, Pernambuco, Brazil; 2 Núcleo de Nutrição, Centro Acadêmico de Vitória, Universidade Federal de Pernambuco, Vitória de Santo Antão, Pernambuco, Brazil; The Ohio State University, UNITED STATES

## Abstract

Considering the current changes in dietary patterns and the increasing prevalence of excess weight throughout the world, several studies have reported insulin resistance, which is a key driver of many chronic diseases, to be an important public health problem in all age groups. Therefore, the aim of the present study was to identify the prevalence and independent predictors of insulin resistance in Brazilian adolescents. A cross-sectional study was conducted with a probabilistic, representative sample of Brazilian adolescents (n = 37,023) who participated in the Study of Cardiovascular Risk in Adolescents. Data were collected on demographic, socioeconomic, lifestyle, anthropometric, and biochemical characteristics as well as antioxidant micronutrient intake (vitamins A, C, E, zinc, and selenium). Insulin resistance was determined using the Homeostatic Model Assessment for Insulin Resistance (HOMA-IR) and classified based on the 75th percentile of the sample distribution. Insulin resistance was detected in 27% of the adolescents and was more prevalent among those aged 12 to 14 years (PR: 1.26 [95%CI: 1.13;1.41]), those residing in the southern and south-eastern regions of the country (PR: 1.47 [95%CI: 1.27;1.70]), those who were physically inactive (PR: 1.12 [95%CI: 1.02;1.23]), and those did not consume alcohol (PR: 1.50 [95%CI: 1.13;1.99]). The prevalence of insulin resistance was 2.5-fold higher among individuals with severe obesity (PR: 2.49 [95%CI: 2.07;3.00]). Waist circumference indicative of cardiovascular risk and high serum triglyceride levels increased the likelihood of insulin resistance (PR: 1.37 [95%CI: 1.19;1.59] and 1.60 [95%CI: 1.45;1.78], respectively). The prevalence of the outcome was higher among adolescents in the lower quartiles of vitamin E intake (p<0.05). In the present study, the prevalence of insulin resistance was high among Brazilian adolescents and we identified sociodemographic, lifestyle, anthropometric, biochemical, and dietary predictors of this outcome.

## Introduction

Hyperinsulinemia and a reduction insulin sensitivity are physiological phenomena inherent to adolescence [1]. However, with the occurrence of the so-called nutritional transition (shift in eating patterns and energy expenditure) and the consequent increase in the prevalence of overweight and obesity in all population groups, insulin resistance (IR) has become a chronic disorder and is considered an important public health problem [2,3].

Previous studies [4,5] recommend the use of the Homeostatic Model Assessment for Insulin Resistance (HOMA-IR) for the identification of IR using cut-off points validated for the population studied. Despite the absence of standardised cut-off points for youths in Brazil [4], studies report that the prevalence of IR ranges from 2.1% to 90.8% among adolescents in different stages of sexual maturity and with different nutritional states [6–9].

The establishment of healthy eating habits [10] and a greater intake of foods with antioxidant properties [11] in childhood is significantly associated with lower adiposity as well as a lower occurrence of IR in adolescence.

The identification of IR is extremely important to the prevention of chronic noncommunicable diseases and can assist in the establishment of strategies for metabolic control in critical phases of development. Therefore, the aim of the present study was to identify the prevalence and independent predictors of IR in Brazilian adolescents.

## Methods

### Study design and population

A national, school-based survey was conducted between February 2013 and November 2014 involving adolescents aged 12 to 17 years enrolled at public and private schools in municipalities with more than 100 thousand residents throughout Brazil. The data used for this study were part of a databank provided by the Federal University of Rio de Janeiro, which was the coordinating institution of the base project: *Estudo de Riscos Cardiovasculares em Adolescentes* (ERICA [Study of Cardiovascular Risk in Adolescents]). This was a national multicentre study with the aim of determining the proportion of Brazilian adolescents with *Diabetes mellitus* and obesity as well as risk factors for cardiovascular disease and markers of IR in this population [12].

This study received approval from the Institutional Review Board (IRB) of the Institute for Collective Health Studies, Federal University of Rio de Janeiro (process number: 45/2008) as well as an IRB in each unit of the federation. The sample comprised only those students from schools at which signed authorisation was obtained from the director. The adolescents who agreed to participate signed a statement of consent and presented a statement of informed consent signed by a legal guardian, when required by the local IRB.

The inclusion of students in the study was determined based on previously defined eligibility criteria [13]: male and female adolescents between 12 and 17 years of age in the last three years of primary school or in high school. The exclusion criteria were physical disability that impeded the anthropometric evaluation, chronic illness (except obesity), the regular use of medications with an effect on blood pressure, blood glucose or lipid metabolism, pregnancy, and endogenous or secondary obesity.

The data collection process involved the administration of questionnaires with approximately 100 questions distributed among 11 topics: sociodemographic characteristics, work, physical activity, diet, smoking, the use of alcoholic beverage, reproductive health, oral health, self-reported morbidities, sleep duration, and common mental disorders. A summarized model of the questionnaire is found in Bloch et al. [12].

For the present study, demographic and socioeconomic characteristics were collected for the characterisation of the sample and statistical adjustments. Anthropometric, biochemical, lifestyle, and dietary (calorie and antioxidant micronutrient intake) characteristics were also collected.

The answers to questions addressing demographic, socioeconomic, and lifestyle characteristics of the adolescents were recorded with the aid of an electronic device (Personal Digital Assistant [PDA], LG GM750Q, LG Electronics, Seoul, South Korea). Information on nutritional status, food intake and blood for the biochemical evaluation were collected by duly trained professionals. The anthropometric data were collected by the team of researchers and recorded in the PDA. All data were simultaneously transferred to the central server of the ERICA study to comprise the databank. The results of the biochemical exams were recorded on spreadsheets under the technical care of the clinical analysis laboratory, set to the central server of the ERICA study, and incorporated into a digital system designed especially for this study.

For the collection of the dietary variables, trained professionals used a specific software program for the direct input of the information on netbooks. The software contained a list of items from the food and beverage databank of the 2008–2009 Family Budget Survey [14,15]. Items not found in the database were added by the interviewers.

### Sampling plan

A representative sample was obtained on the national, regional, and municipal levels and involved three selection stages (school, class, and students) with probability proportional to the universe. At the selected schools, a survey was conducted of the classes and students to enable the inclusion of three classes per school. All students in the selected classes were invited to participate in the study [12].

The sample size was calculated considering a 4% rate of metabolic syndrome, a maximum error of 0.9%, and a 95% confidence level. The minimum size for a simple random sample was determined to be 1,821 students. As cluster sampling was performed by school, class, and school year, a design effect of 2.97 was considered for mean body mass obtained from the 2007 survey of the Surveillance System for Risk Factors Affecting Adolescent Health implemented by the City of Rio de Janeiro, Brazil [12,13]. Fifteen percent was added to the sample size to compensate for possible dropouts, resulting in 6,219 adolescents. As the study should produce estimates with precision specified for each of the 12 domains (six ages x two sexes), this led to a total sample of 74,628 adolescents, which was rounded up to 75,060 adolescents, since multiple samples of 60 adolescents were needed for each stratum [13].

As the collection of the blood for the biochemical analysis required 12 hours of fasting, this evaluation was performed exclusively with individuals enrolled in morning classes. Therefore, the final sample was composed of 37,023 adolescents. Further information on the sampling and technical procedures is available in previous publications [12,13].

### Demographic and socioeconomic characteristics

Demographic and socioeconomic characteristics (sex, skin colour, and mother’s schooling) were collected during interviews with the participants. The Brazilian Economic Classification System was used for the determination of economic class. This instrument employs a point system based on the possession of household goods and schooling of the head of the family for the classification of economic status in the following categories: Upper class (subcategories A1 and A2), middle class (subcategories B1, B2, and C1), and lower class (subcategories C2, D, and E).

### Anthropometric variables

Weight, height, and waist circumference (WC) were measured. Weight was determined on a digital scale with a capacity of 200kg and precision of 50g. Height was measured in duplicate using a portable stadiometer with a precision of 0.1cm (permitting a maximum variation of 0.5cm between the two readings, followed by the calculation of the mean). For the determination of weight and height, the adolescents stood barefoot wearing light clothing [12]. WC was measured using a nonflexible metric tape with a precision of 0.1cm. For this measurement, the adolescent stood with the abdomen relaxed, arms alongside the body, feet together with body weight equally distributed between both legs. The metric tape was placed horizontally at the midpoint between the lower edge of the last rib and the iliac crest [12].

Nutritional status was classified using the Anthro software program (2007) considering body mass index for age (BMI/A) expressed in z-scores. The reference standard for the categorisation of the measures of weight and height was that recommended by the World Health Organization (WHO) [16] and Brazilian Health Ministry.^(16)^ Adolescents with BMI/A z-score < -2 were classified as underweight, those with z-score ≥ -2 and ≤ +1 were considered to be in the ideal range (normal weight), those with z-score > +1 and ≤ +2 were classified as overweight, and those with z-score > +2 or > +3 were identified as having obesity or severe obesity, respectively.

The cut-off points used for the determination of cardiovascular risk based on the WC were those recommended by Freedman et al. [17], who identified WC ≥ the 90^th^ percentile as indicative of high risk. WC and height were used for the calculation of the waist-to-height (W/Ht) ratio, considering ≥ 0.5 as the cut-off point for abdominal obesity [18].

### Biochemical variables

The biochemical analysis provided data on fasting blood sugar, fasting insulinemia, and the lipid profile. The adolescents and the guardians were informed about the need for fasting 12 hours before the collection of the blood on the scheduled day. Blood collection was the responsibility of a clinical analysis laboratory and was performed by trained professionals. Prior to the collection, the adolescents were interviewed to verify that they had fasted. The blood collection was performed at the schools in a standardised manner and the samples were analysed at a single laboratory. The blood was collected through a vein puncture using disposable material and a stored in a 5-mL tube kept in ice [19].

Plasma glucose was evaluated using the GOD-PAP enzyme method with the aid of the Roche modular analytical equipment. Plasma insulin was evaluated using the preferred immunometric methods at the analysis laboratory due to the greater sensitivity and specificity. The lipidogram included the determination of total cholesterol, HDL-c, and triglycerides (TG), which were analysed using the colorimetric method with the aid of the Roche modular analytical equipment. LDL-c was calculated using the following equation: LDL-c = total cholesterol total—(HDL-c + TG/5). The reference values listed in the First Brazilian Guidelines for the Prevention of Atherosclerosis in Childhood and Adolescence [20] were used for the classification of the serum lipids.

### Determination of insulin resistance

The HOMA-IR index was determined from the fasting glucose and insulinemia values using the pre-established formula: HOMA-IR = (fasting insulinemia x fasting glucose)/22.5 [21]. Due to the absence of standardisation regarding the cut-off points for the determination of IR in adolescents using the HOMA-IR index, the 75^th^ percentile (P_75_) was considered using the distribution of the sample stratified by sex and stage of sexual maturation [22–25]. The P_75_ values of the HOMA-IR index for the different groups are displayed in Table 1.

**Table 1:** 75^th^ percentile of Homeostatic Model Assessment for insulin resistance according to sexual maturation stage. ERICA study, 2013–2014.

P_75_ of HOMA-IR for pubescent boys and girls	P_75_ of HOMA-IR for post-pubescent boys and girls
Pubescent boys = 2.27	Post-pubescent boys = 2.20
Pubescent girls = 2.48	Post-pubescent girls = 2.59

P_75_ = 75^th^ percentile of distribution; HOMA-IR = Homeostatic Model Assessment for Insulin Resistance.

### Dietary intake

Dietary intake was determined using the 24-hour recall (24hR) technique and employing the Multiple Pass Method with the aid of the ERICA-REC24 software, which was designed especially for the study [12]. Energy and nutrient intake was determined using the Nutritional Composition Table of Foods Consumed in Brazil [14] and the Table of Reported Measures for Foods Consumed in Brazil [15]. For the purposes of analysis, intake quartiles of the following antioxidant micronutrients were considered: vitamins A, C, E, zinc, and selenium. Energy intake (calories) was also used for the statistical adjustments.

### Sexual maturation

The stage of sexual maturation was self-reported by the adolescents using the indicative figures standardised by Tanner (1991) in "Growth at adolescence". Based on the analysis of the figures, the participants were divided into sexual maturation categories: Stage I = pre-pubescent; Stages II, III, and IV = pubescent; Stage V = post-pubescent. As the sample included adolescents beginning at 12 years of age, the percentage of pre-pubescent individuals was low. Therefore, to enhance the analytical power of the study, this group of individuals was included in the pubescent category to enable the dichotomisation of sexual maturation as pubescent or post-pubescent.

### Lifestyle

For data on the use of cigarettes and alcohol, the monthly frequency of the consumption of these drugs was considered based on the recommendations of the Youth Risk Behavior Survey of the US Centers for Disease Control [26]. Smoking was defined as the consumption of one or more cigarettes in the previous 30 days. Binge drinking was established as the consumption of five or more alcoholic beverages on a single occasion in the previous 30 days.

Physical activity level was determined using the International Physical Activity Questionnaire (IPAQ) [27]. Adolescents who reported participating in at least 60 minutes of moderate to vigorous physical activities five or more days per week were considered active and the others were considered insufficiently active.

### Statistical analysis

The ERICA study had a complex sample as a result of the stratification, clustering, and unequal probabilities during the different stages of the selection process [13]. Therefore, the analyses were performed using the Survey (svy) module of STATA software, version 14.0, which enables adjusting for a complex sampling design.

For the association analyses, the explanatory variables were grouped hierarchically beginning with biological factors, followed by I) socioeconomic factors, II) lifestyle factors, III) dietary factors, and IV) nutritional (anthropometric) and metabolic (biochemical) factors. The model was created considering the theoretical basis of factors associated with the development of IR, which implies different hierarchical levels of determination.

For the determination of associations between IR and the independent variables, univariate analysis was performed for each level of determination using Pearson’s chi-square test. Next, Poisson regression analysis with robust variance was used to investigate how the prevalence of IR was influenced by the explanatory variables. To determine the risk effect on IR, the category with the lowest prevalence of the outcome was considered the reference category. Using the stepwise forward method, the variables on the first hierarchical level were analysed together and those with a p-value ≥ 0.20 were excluded one by one, depending on the pre-established effect on the determination of the outcome. Variables that exhibited collinearity were sequentially excluded from the model. The variables on the second hierarchical level were then incorporated into the model, proceeding in the same manner for all subsequent levels. The results of the association analyses were expressed as prevalence ratios (PR) and respective 95% confidence intervals (95%CI). A p-value ≤ 0.05 was considered statistically significant.

## Results

The sample of 37,023 adolescents was representative of 6,628,961 Brazilian adolescents. The sample was predominated by individuals residing in the southern and south-eastern regions (65%), those residing in cities other than state capitals (58.1%), and those enrolled at public schools (77.7%) (Table 2).

**Table 2:** General characterisation of Brazilian adolescent students. ERICA Study, 2013–2014.

Variables	Sample	Population	%	95%CI
**Regional Distribution**				
North/Northeast	18,509	1,794,092	27.1	26.9–27.2
South/Southeast	13,098	4,309,529	65.0	64.8–65.1
Midwest	5,416	525,340	7.9	7.7–8.0
**Geographic Stratum**				
Capital city	27,350	2,777,952	41.9	41.7–42.1
Interior	9,673	3,851,009	58.1	57.9–58.2
**Type of School**				
Public	27,268	1,477,670.8	77.7	72.4–82.2
Private	9,755	5,151,290.1	22.3	17.7–27.6
**Sex**				
Male	14,811	3,304,088	49.8	49.7–49.9
Female	22,212	3,324,873	50.2	50.0–50.3
**Age Group**				
12–14 years	16,959	3,089,012	46.6	46.4–46.7
15–17 years	20,064	3,539,949	53.4	53.2–53.5
**Sexual Maturation**				
Stage I	168	30,984.5	0.5	0.3–0.5
Stage II	1,857	366,356.8	5.0	4.8–5.2
Stage III	6,506	1,124,767.8	17.6	17.1–17.9
Stage IV	14,550	2,640,945.9	39.3	38.8–39.8
Stage V	13,919	2,465,905.8	37.6	37.1–38.1
**Skin Colour**				
White	13,255	2,691,957.7	40.6	38.7–42.5
Non-White	22,937	3,937,003.2	59.4	57.4–61.2
**Mother’s Schooling**				
≤ 8 years	8,822	1,725,931.6	26.1	23.4–28.9
> 8 years	20,019	3,208,220.9	48.4	44.8–51.9
Does not know/remember	8,182	1,694,808.4	25.5	24.0–27.1
**Economic Status**^a^				
Upper class	3,419	517,357.4	7.8	6.7–9.0
Middle class	19,657	3,503,950.0	52.8	51.4–54.3
Lower class	13,947	2,607,653.5	39.4	37.7–40.9
**Physical Activity Level**				
Active	17,170	3,485,367.6	52.6	51.4–53.6
Inactive	17,373	3,143,593.4	47.4	46.3–48.5
**Alcohol Consumption**				
Yes	7,541	1,430,031.1	21.6	20.3–22.8
No	28,264	4,949,387.3	74.6	73.3–75.9
Does not know/remember	1,218	249,542.5	3.8	3.2–4.4
**Smoking**				
Yes	1,386	291,848.5	4.4	3.9–4.9
No	35,416	6,287,196.6	94.8	94.2–95.3
Does not know/remember	221	49,915.8	0.8	0.5–1.0

95%CI: 95% confidence interval.

^a^Upper class = subcategories A1-A2; Middle class = B1-C1; Lower class = C2-E [15].

Regarding nutritional status, 27% of the sample was classified with excess weight and distributed among the categories overweight, obesity, and severe obesity. Moreover, 11.6 and 14.4% exhibited high cardiovascular risk and accumulated fat in the abdominal region according to the WC and W/Ht ratio, respectively (Table 3). A larger portion of the sample had desirable levels of TG, HDL-C, LDL-C, and total cholesterol, although substantial percentages of patients with threshold or high levels of these variables were found (Table 3).

**Table 3:** Description of anthropometric and biochemical characteristics of Brazilian adolescent students. ERICA Study, 2013–2014.

Variables	Sample	Population	%	95%CI
**BMI/Age**^a^				
Underweight	990	148,796.3	2.3	1.9–2.6
Normal weight	26,356	4,690,101.4	70.7	69.1–72.3
Overweight	6,588	1,171,379.6	17.7	16.5–18.8
Obesity	2,702	546,387.8	8.2	7.5–8.9
Severe obesity	387	72,295.7	1.1	0.8–1.3
**Waist Circumference**^b^				
Low risk	33,266	5,857,313.9	88.4	87.3–89.3
High risk	3,699	771,647.1	11.6	10.7–12.6
**Waist/Height Ratio**^c^				
Without abdominal obesity	31,925	5,674,688.7	85.6	84.5–86.6
With abdominal obesity	5,040	954,272.3	14.4	13.4–15.4
**Triglycerides**				
Desirable	29,564	5,319,192.9	80.2	78.8–81.5
Borderline	4,428	792,422.2	12.0	11.0–12.9
High	2,924	517,345.8	7.8	7.1–8.5
**HDL-Cholesterol**				
Desirable	19,739	3,542,161.3	53.4	51.3–55.5
Undesirable	17,179	3,086,799.7	46.6	44.4–48.7
**LDL-Cholesterol**				
Desirable	28,028	5,094,279.5	76.8	75.7–77.9
Borderline	7,473	1,295,954.7	19.6	18.5–20.5
**LDL-Cholesterol**				
High	1,403	238,726.7	3.6	3.1–4.0
**Cholesterol Total**				
Desirable	20,246	3,677,543.6	55.5	53.5–57.4
Borderline	9,065	1,607,707.7	24.2	22.7–25.8
High	7,607	1,343,709.7	20.3	19.0–21.5

95%CI: 95% confidence interval; BMI/Age: Body mass index for age.

^a^Classified as underweight (z-score<-2), normal weight (z-score≥-2 ≤+1), overweight (z-score>+1≤+2), obesity (z-score>+2), and severe obesity (z-score>+3).

^b^High risk when ≥90^th^ percentile [18].

^c^Indicative of abdominal obesity when ≥0.5 [19].

**Fig 1:**
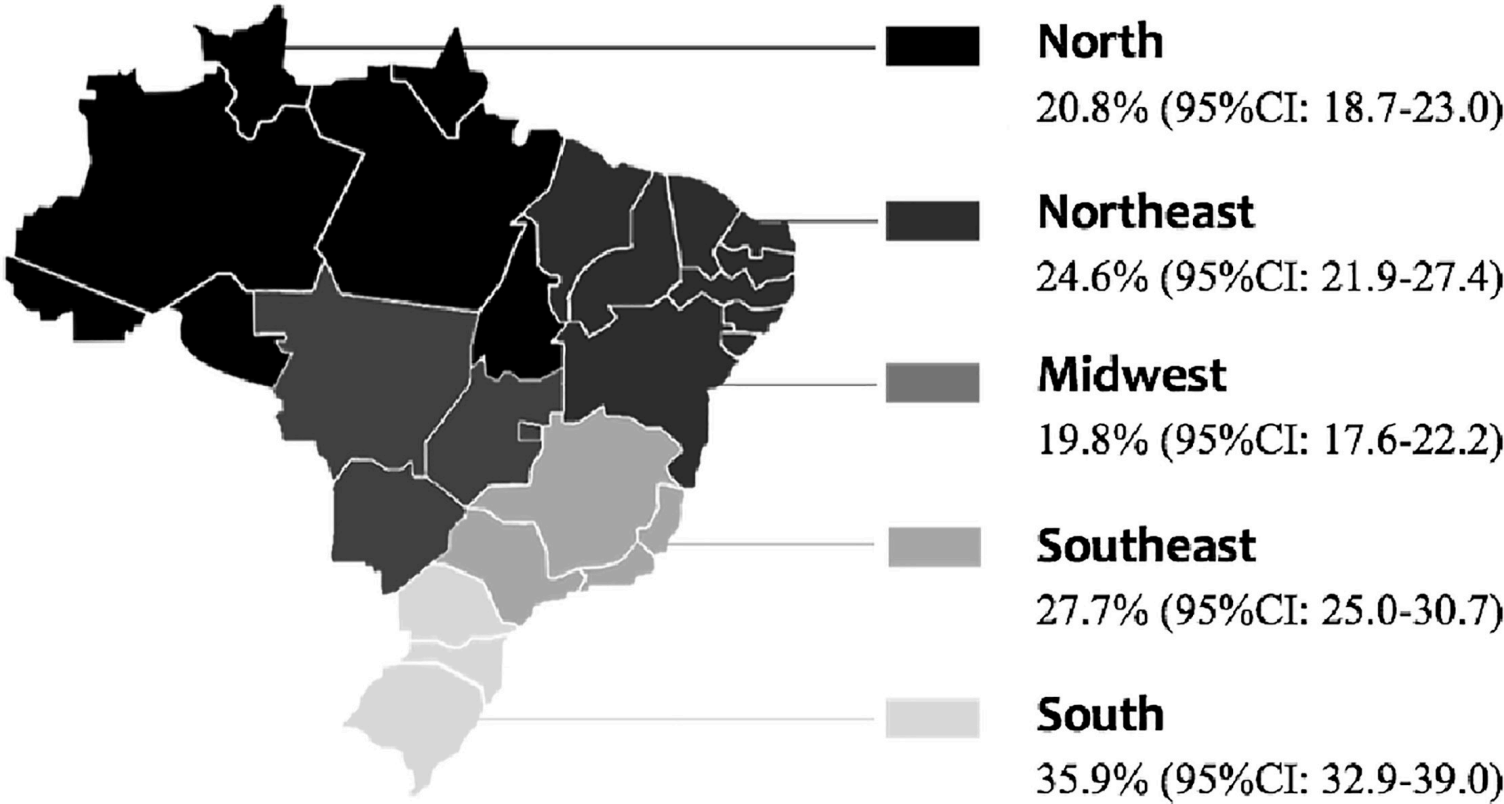
Prevalence of insulin resistance in Brazilian adolescents according to regional distribution. ERICA Study, 2013–2014. 95%CI: 95% Confidence Interval. Source: The authors.

IR was detected in 27% of the population, with the highest percentage found in the southern region (35.9%) and the lowest found in the midwestern region (19.8%). Fig 1 displays the distribution of IR among the different regions of Brazil.

The investigation of associations between IR and the independent variables followed a pre-established hierarchical model for the input of variables. Considering Levels I and II (socioeconomic and lifestyle characteristics), the likelihood of IR was higher among adolescents residing in the southern/south-eastern regions, those between 12 and 14 years of age, those with self-declared white skin, those classified as physically inactive, and those who did not consume alcohol or tobacco (Table 4).

**Table 4:** Prevalence of insulin resistance, prevalence ratios, and confidence intervals according to demographic, socioeconomic, and lifestyle characteristics of Brazilian adolescent students. ERICA Study, 2013–2014.

Independent Variables	Insulin resistance		p-value*
	% (95%CI)	PR (95%CI)	
**Regional Distribution**			
Midwest	19.8 (17.6–22.2)	*Ref*.	**<0.001**
North/Northeast	23.6 (21.6–25.8)	1.19 (1.03;1.38)	
South/Southeast	28.3 (26.9–31.8)	1.48 (1.28;1.70)	
**Sex**			
Male	26.1 (23.9–28.5)	*Ref*.	0.246
Female	27.9 (25.4–28.7)	1.06 (0.95;1.19)	
**Age Group**			
15–17 years	24.2 (22.3–26.2)	*Ref*.	**<0.001**
12–14 years	30.2 (27.8–32.8)	1.24 (1.12;1.39)	
**Sexual Maturation**			
Stage I	29.7 (18.3–44.2)	1.31 (0.81;2.10)	0.361
Stage II	22.6 (19.1–26.5)	*Ref*.	
Stage III	27.8 (24.6–31.2)	1.22 (1.01;1.50)	
Stage IV	27.2 (25.0–29.5)	1.20 (1.01;1.44)	
Stage V	27.1 (25.0–29.2)	1.19 (1.01;1.42)	
**Skin Colour**			
Non-white	25.7 (24.0–27.5)	*Ref*.	**0.004**
White	28.5 (26.4–30.7)	1.10 (1.03;1.18)	
**Mother’s Schooling**			
> 8 years	26.1 (24.3–27.9)	*Ref*.	0.361
≤ 8 years	27.6 (25.0–30.3)	1.05 (0.94;1.17)	
Does not know/remember	28.1 (25.1–31.4)	1.07 (0.95;1.21)	
**Economic Status**^a^			
Upper class	27.6 (22.8–33.1)	1.04 (0.84;1.28)	0.808
Middle class	26.6 (24.4–28.9)	*Ref*.	
Lower class	27.5 (25.2–29.9)	1.03 (0.92;1.15)	
**Physical Activity Level**			
Active	25.5 (23.6–27.5)	*Ref*.	**0.009**
Inactive	29.0 (26.7–31.4)	1.13 (1.03;1.24)	
**Alcohol consumption**			
Does not know/remember	15.0 (8.9–24.1)	*Ref*.	0.139
Yes	25.5 (19.5–32.5)	1.69 (0.93;3.08)	
No	27.2 (25.6–28.9)	1.81 (1.09;2.99)	
**Smoking**			
Does not know/remember	19.7 (15.2–25.0)	*Ref*.	**0.001**
Yes	24.5 (21.7–27.6)	1.24 (0.98;1.58)	
No	28.1 (26.4–29.8)	1.42 (1.12;1.82)	

95%CI: 95% confidence interval; PR: Prevalence ratio.

*x^2^ test.

^a^Upper class = subcategories A1-A2; Middle class = B1-C1; Lower class = C2-E [15].

On Level III, all antioxidant micronutrients, except selenium, were significantly associated with IR (Table 5).

**Table 5:** Prevalence of insulin resistance, prevalence ratios, and confidence intervals according to antioxidant micronutrient intake by Brazilian adolescent students. ERICA Study, 2013–2014.

Independent Variables	Insulin resistance		p-value*
	% (95%CI)	PR (95%CI)	
**Zinc**			
< 7.5 mg	30.3 (27.7–32.9)	1.23 (1.10;1.38)	**0.002**
7.5–10.9 mg	27.9 (25.2–30.8)	1.14 (1.01;1.28)	
10.9–16.3 mg	25.3 (22.8–28.0)	1.03 (0.93;1.14)	
> 16.3 mg	24.4 (22.3–26.6)	*Ref*.	
**Selenium**			
< 53.3 μg	27.2 (24.0–30.6)	1.08 (0.94;1.24)	0.463
53.3–81.8 μg	28.2 (24.7–32.1)	1.12 (0.99;1.26)	
81.8–125.3 μg	27.4 (25.4–29.5)	1.08 (0.95;1.24)	
> 125.3 μg	25.2 (22.7–27.7)	*Ref*.	
**Retinol**			
< 95.8 μg	28.6 (26.5–30.7)	1.19 (1.08;1.30)	**0.024**
95.8–185.7 μg	28.4 (26.0–30.9)	1.18 (1.05;1.32)	
185.7–315.8 μg	26.9 (23.8–30.1)	1.11 (0.97;1.28)	
> 315.8 μg	24.0 (21.8–26.4)	*Ref*.	
**Vitamin C**			
< 11.2 mg	27.9 (25.5–30.4)	1.16 (1.03;1.30)	**0.022**
11.2–50.2 mg	27.8 (26.0–29.8)	1.15 (1.01;1.32)	
50.2–123.2 mg	28.1 (25.8–30.5)	1.17 (1.04;1.31)	
>123.2 mg	24.0 (21.2–27.1)	*Ref*.	
**Vitamin E**			
< 2.4 mg	29.8 (27.2–32.6)	1.29 (1.12;1.49)	**<0.001**
2.4–3.7 mg	30.4 (27.7–33.1)	1.31 (1.17;1.48)	
3.7–5.4 mg	25.5 (23.3–27.7)	1.10 (0.97;1.25)	
>5.4 mg	23.0 (20.5–25.8)	*Ref*.	

Antioxidant micronutrient intake expressed in quartiles of sample distribution. 95%CI: 95% confidence interval; PR: Prevalence ratio; Ref = reference (1.0).

*x^2^ test.

The anthropometric and biochemical characteristics (Level IV) were also significantly associated with the outcome (Table 6).

**Table 6:** Prevalence of insulin resistance, prevalence ratios, and confidence intervals according to anthropometric and biochemical characteristics of Brazilian adolescent students. ERICA Study, 2013–2014.

Independent Variables	Insulin resistance		p-value*
	% (95%CI)	PR (95%CI)	
**BMI/Age**^a^			
Without excess weight	18.2 (16.7–19.7)	*Ref*.	**<0.001**
Overweight	40.0 (37.5–42.6)	2.20 (2.04;2.36)	
Obesity	69.6 (65.3–73.6)	3.82 (3.47;4.21)	
Severe obesity	86.8 (79.9–91.6)	4.77 (4.32;5.26)	
**Waist Circumference**^b^			
Low risk	21.6 (20.0–23.1)	*Ref*.	**<0.001**
High risk	68.2 (64.1–72.1)	3.16 (2.89;3.45)	
**Waist/Height Ratio**^c^			
Without abdominal obesity	20.6 (19.2–22.1)	*Ref*.	**<0.001**
With abdominal obesity	64.9 (61.5–68.1)	3.14 (2.92;3.38)	
**Triglycerides**			
Desirable	22.5 (20.9–24.1)	*Ref*.	**<0.001**
Borderline	41.0 (37.2–45.0)	1.82 (1.65;2.00)	
High	52.6 (47.7–57.5)	2.34 (2.09;2.61)	
**HDL-Cholesterol**			
Desirable	23.1 (21.3–24.9)	*Ref*.	**<0.001**
Undesirable	31.5 (29.6–33.5)	1.36 (1.27;1.45)	
**LDL-Cholesterol**			
Desirable	25.4 (23.5–27.5)	*Ref*.	**<0.001**
Borderline	31.5 (29.3–33.7)	1.23 (1.12;1.36)	
High	36.3 (30.7–42.4)	1.42 (1.21;1.68)	
**Cholesterol Total**			
Desirable	24.7 (22.9–26.6)	*Ref*.	**<0.001**
Borderline	27.5 (24.5–30.8)	1.11 (0.99;1.23)	
High	32.6 (30.1–35.1)	1.31 (1.20;1.43)	

95%CI: 95% confidence interval; PR: Prevalence ratio; Ref = reference (1.0).

*x^2^ test.

BMI/Age: Body mass index for age.

^a^Classified as underweight (z-score<-2), normal weight (z-score≥-2 ≤+1), overweight (z-score>+1≤+2), obesity (z-score>+2), and severe obesity (z-score>+3).

^b^High risk when ≥90^th^ percentile [18].

^c^Indicative of abdominal obesity when ≥0.5 [19].

After the statistical adjustments, IR was more prevalent among adolescents residing in the southern/south-eastern regions, those between 12 and 14 years of age, those classified physically inactive, and those who did not consume alcohol. The prevalence of IR was approximately 2.5-fold higher among individuals with severe obesity (PR: 2.49 [95%CI: 2.07;3.00]) compared to other classifications of BMI/A. The prevalence of IR was also higher among adolescents with cardiovascular risk determined by high WC (PR: 1.37 [95%CI: 1.19;1.59]) and TG values (PR: 1.60 [95%CI: 1.45;1.78]) (Table 7).

**Table 7:** Crude and adjusted prevalence ratios for effects of explanatory variables on insulin resistance in Brazilian adolescents. ERICA Study, 2013–2014.

Independent Variables	Insulin resistance				p-value*
	Crude Analysis		Adjusted Analysis		
	PR	95%CI	PR	95%CI	
**Age Group**					
15–17 years	*Ref*.		*Ref*.		
12–14 years	1.24	(1.12;1.39)	1.26	(1.13;1.41)	**<0.001**
**Regional Distribution**					
Midwest	*Ref*.		*Ref*.		
North/Northeast	1.19	(1.03;1.38)	1.18	(1.02;1.37)	**0.024**
South/Southeast	1.48	(1.28;1.70)	1.47	(1.27;1.70)	**<0.001**
**Physical Activity Level**					
Active	*Ref*.		*Ref*.		
Inactive	1.13	(1.03;1.24)	1.12	(1.02;1.23)	**0.010**
**Alcohol consumption**					
Does not know/remember	*Ref*.		*Ref*.		
Yes	1.69	(0.93;3.08)	1.34	(1.02;1.77)	**0.034**
No	1.81	(1.09;2.99)	1.50	(1.13;1.99)	**0.005**
**Vitamin E**					
< 2.4 mg	1.29	(1.12;1.49)	1.26	(1.07;1.49)	**0.005**
2.4–3.7 mg	1.31	(1.17;1.48)	1.29	(1.14;1.47)	**<0.001**
3.7–5.4 mg	1.10	(0.97;1.25)	1.09	(0.95;1.26)	0.218
> 5.4 mg	*Ref*.		*Ref*.		
**BMI/Age**^a^					
Without excess weight	*Ref*.		*Ref*.		
Overweight	2.20	(2.04;2.36)	1.88	(1.69;2.09)	**<0.001**
Obesity	3.82	(3.47;4.21)	2.21	(1.88;2.60)	**<0.001**
Severe obesity	4.77	(4.32;5.26)	2.49	(2.07;3.00)	**<0.001**
**Waist Circumference**^b^					
Low risk	*Ref*.		*Ref*.		
High risk	3.16	(2.89;3.45)	1.37	(1.19;1.59)	**<0.001**
**Triglycerides**					
Desirable	*Ref*.		*Ref*.		
Borderline	1.82	(1.65;2.00)	1.46	(1.34;1.60)	**<0.001**
High	2.34	(2.09;2.61)	1.60	(1.45;1.78)	**<0.001**

95%CI: 95% confidence interval; PR: Prevalence ratio; Ref = reference (1.0).

*Poisson regression with robust variance (Level I adjusted by sexual maturation stage; Level II adjusted by Level I and sexual maturation stage; Level III adjusted by Level I and II, sexual maturation stage, and energy intake; Level IV adjusted by Levels I, II, and III, sexual maturation stage, and energy intake).

BMI/Age: Body mass index for age.

^a^Classified as underweight (z-score<-2), normal weight (z-score≥-2 ≤+1), overweight (z-score>+1≤+2), obesity (z-score>+2), and severe obesity (z-score>+3).

^b^High risk when ≥90^th^ percentile [18].

Among the antioxidant micronutrients, the association with vitamin E was maintained in the multivariate analysis (Table 7). IR was more prevalent among adolescents in the lowest quartiles of vitamin E intake.

## Discussion

Using the P_75_ of the distribution of the HOMA-IR index in the present study, 27% of the sample of Brazilian adolescents exhibited IR and the prevalence was higher among individuals living in the southern region of the country (35.9%).

Due to the lack of a consensus on cut-off points of the HOMA-IR for the determination of this outcome in adolescents [4,5], studies estimating the prevalence of IR in this population group report different percentages depending on the cut-off point used as the diagnostic criterion [28–31]. In the present investigation, the HOMA-IR index was adjusted for sex and sexual maturation stage in order to identify cut-off points more in line with the stage of life of the adolescent at the time of the study. Previous studies using similar strategies found cut-off points ranging from 3.77 to 4.52 for male and female Brazilian and Hispanic adolescents in different sexual maturation stages [23,24]. According to Shashaj et al. [25], the analysis of the percentage distribution of the HOMA-IR index is a useful tool in clinical practice and P_75_ is an accurate value for the suspicion of the occurrence of cardiometabolic risk factors in adolescents, such as abnormal serum lipid and glucose levels.

The higher prevalence of IR among adolescents residing in the southern region may be secondary to the dietary patterns found in this geographic region, which are characterised by the high consumption of ultra-processed foods (soft drinks, sweets, and cheeses) rich in simple carbohydrates, saturated fat, and sodium [32]. This finding reflects the higher prevalence of excess weight among adolescents in the region, as described in the 2008–2009 Family Budget Survey [33], which found that 24.6% of individuals between 10 and 19 years of age had excess weight.

The present data demonstrate that younger adolescents (12 to 14 years) were more likely to have IR and this association remained significant after the statistical adjustment. According to Kelsey and Zeitler [34], the prevalence of IR is generally higher among girls in the age group corresponding to sexual maturation stages II and III (classified as pubescent). Although we found a greater percentage of girls in sexual maturation stage III in the present study, no significant difference in the prevalence of IR was found between boys and girls. The general predominance of this disorder in girls may be explained by the fact that sex hormones affect girls at an earlier age than boys [28]. Thus, there is a greater stimulus of body remodelling and consequent deposition of fat in the visceral region [2,35,36], which contributes to the development of IR in the initial stages of adolescence among girls.

Regarding the practice of physical exercise, sedentary adolescents had higher frequencies of IR. In the adjusted analysis, the prevalence of IR was 1.12-fold (95%CI: 1.02;1.23) higher among adolescents classified as inactive. A meta-analysis conducted by Fedewa et al. [37] confirms the fact that exercise is capable of lowering blood sugar levels and improving IR in children and adolescents. Physical activity seems to exert an influence on sensitivity to insulin, as it is capable of enhancing the transport of GLUT4-dependent glucose and stimulating the loss of adipose tissue [38]. In a randomised trial with 40 female adolescents, the combined practice of aerobic and resistance exercises led to a significant improvement in HOMA-IR (3.3±0.2 *vs*. 1.9±0.1; p<0.05), which occurred concomitantly with weight loss secondary to the intervention [39].

The consumption of alcohol and tobacco was also associated with IR. However, the association with smoking lost its significance in the adjusted analysis. A significantly higher frequency of IR was found among the adolescents who did not drink alcohol. However, this effect may have been due to the greater percentage of individuals classified as non-drinkers in the overall sample (74.6%).

In the analysis of antioxidant micronutrient intake, all micronutrients, except selenium, were associated with IR in the crude analysis. After the adjustments for energy intake and the hierarchical levels established for the multivariate analysis, only vitamin E remained associated with the outcome, as the individuals in the first (PR = 1.26; 95%CI: 1.07;1.49) and second (PR = 1.29; 95%CI: 1.14;1.47) quartiles (lower intake of this vitamin) had a greater chance of exhibiting IR. A population-based cohort study conducted by Montonen et al. [40] demonstrated the vitamin E intake in adults was inversely proportional to type 2 *Diabetes mellitus*, reporting a relative risk of 0.69 (95%CI: 0.51;0.94) in the highest intake quartile for this vitamin (>P_75_). However, a meta-analysis of randomised clinical trials conducted by Xu et al. [41] reported that there is insufficient evidence to propose the supplementation of vitamin E as a means of glycaemic control, which underscores the need to encourage food sources through dietary planning. Vitamin E has important effects on glycaemic homeostasis, reducing the generation of advanced glycation end products, attenuating the dysfunction of pancreatic ß-cells [41], and reducing free radicals and the oxidation of LDL-c when combined with vitamin C [42].

Regarding the lipid profile, we found substantial percentages of individuals with borderline and high levels of LDL-c, TG, and total cholesterol as well as low levels of HDL-c. Cunha et al. [43] analysed the lipid profile of 600 adolescents (10 to 19 years of age) in the state of Paraná (south Brazil) and found altered lipid levels, with a similar proportion of high LDL-c (23%), lower proportion of high total cholesterol (28%), and higher proportions of low HDL-c (52%) and borderline or high TG (30%) in comparison to the frequencies found in the present investigation.

In the adjusted analysis of the lipid variables, only TG was associated with IR. It seems that TG levels increase with the advance of puberty in both sexes. In a cohort study following up America children through to adolescence, Perng et al. [44] found a mean TG level of 57.6 ± 23.5 at baseline (mean age in childhood: 7.9±0.8 years) and 69.4±30.6 at follow-up (mean age in adolescence: 13.1±0.9 years) (p<0.001). This finding is similar to the insulin changes found in the pubescent adolescents, which contributed to the association between these variables.

Regarding nutritional status, adolescents with excess weight (overweight to severe obesity) had substantial frequencies of IR. The likelihood of IR in the adjusted analysis was threefold greater among those diagnosed with severe obesity (PR = 2.29; 95%CI: 2.07;3.00). Both WC and the W/Ht ratio were associated with IR in the crude analysis, but only WC remained associated after the statistical adjustments.

Excess weight is one of the aggravating factors of IR. In a study involving Chilean children and adolescents (n = 208), a significant increase in mean HOMA-IR index values was found as excess weight (determined based on BMI/A) became more extreme (p<0.001) [45].

Evaluating 1,125 children and adolescents (five to 19 years of age) at public and private schools in the municipality of Uberaba in the state of Minas Gerais (south-eastern Brazil), Palhares et al. [46] found significant correlations between the HOMA-IR index and both the BMI z-score and abdominal circumference (p<0.001) as well as correlations with other metabolic variables associated with cardiovascular risk. Similarly, Morais et al. [1] found that the HOMA-IR index was significantly associated with BMI in normotensive adolescents (r = 0.366, p<0.031) and those with altered blood pressure (r = 0.394, p<0.001); the authors also described correlations with WC in both groups (adolescents with altered blood pressure: r = 0.345, p<0.001; normotensive adolescents: r = 0.345, p = 0.042). The results of a clinical trial conducted by Son et al. [39] with 40 female adolescents indicate that a reduction in WC by approximately 2.2cm promotes an substantial reduction in the HOMA-IR index in this population group. The authors suggest that studies with larger, more robust samples could confirm this finding.

The representative sample in this study enabled the determination of the prevalence and independent predictors of IR among Brazilian adolescents. Despite the strategies employed in the adjusted analysis to control for possible confounding variables and collinearity, some limitations should be addressed for greater clarification of the present results. The cross-sectional design does not enable the establishment of causality. The lack of standardised cut-off points for the HOMA-IR index hinders comparisons with the results of previously conducted studies. The administration of the IPAQ for the determination of physical activity level may constitute a source of bias, as this instrument may overestimate the practice of physical exercise. The scarcity of studies reporting antioxidant micronutrient intake in adolescents was another limiting factor. Moreover, the use of only the 24hR method as a measure of food intake impedes the extrapolation of the dietary data, as this method does not necessary reflect habitual food intake and may therefore not be related to the outcome. However, this was minimised by the use of crude nutrient intake and intake quartiles adjusted for energy intake in the multivariate analysis.

In conclusion, the prevalence of insulin resistance is high among Brazilian adolescents and is associated with sociodemographic, lifestyle, dietary, anthropometric, and biochemical variables. The results of the present study underscore the need for public health measures as well as dietary and nutritional education strategies to promote sustainable eating habits beginning early in life, respecting cultural, social, and environmental aspects for the maintenance of health and the prevention of chronic diseases in Brazilian adolescents.
